# Metabolomic associations of impaired awareness of hypoglycaemia in type 1 diabetes

**DOI:** 10.1038/s41598-024-55032-6

**Published:** 2024-02-23

**Authors:** R. D. M. Varkevisser, A. Cecil, C. Prehn, D. Mul, H. J. Aanstoot, A. D. Paterson, B. H. R. Wolffenbuttel, M. M. van der Klauw

**Affiliations:** 1 Department of Endocrinology, University of Groningen, University Medical Center Groningen, Groningen, The Netherlands; 2 Metabolomic and Proteomics Core, Helmholtz Zentrum München, German Research Center for Environmental Health (GmbH), Neuherberg, Germany; 3 Diabeter Netherlands, Center for Type 1 Diabetes Care and Research, Rotterdam, The Netherlands; 4 Genetics and Genome Biology Program, The Hospital for Sick Children, Toronto, Canada; 5 Divisions of Epidemiology and Biostatistics, Dalla Lana School of Public Health, University of Toronto, Toronto, Canada

**Keywords:** Molecular medicine, Diabetes, Type 1 diabetes, Metabolomics, Genome-wide association studies

## Abstract

This study investigates impaired awareness of hypoglycaemia (IAH), a complication of insulin therapy affecting 20–40% of individuals with type 1 diabetes. The exact pathophysiology is unclear, therefore we sought to identify metabolic signatures in IAH to elucidate potential pathophysiological pathways. Plasma samples from 578 individuals of the Dutch type 1 diabetes biomarker cohort, 67 with IAH and 108 without IAH (NAH) were analysed using the targeted metabolomics Biocrates AbsoluteIDQ p180 assay. Eleven metabolites were significantly associated with IAH. Genome-wide association studies of these 11 metabolites identified significant single nucleotide polymorphisms (SNPs) in C22:1-OH and phosphatidylcholine diacyl C36:6. After adjusting for the SNPs, 11 sphingomyelins and phosphatidylcholines were significantly higher in the IAH group in comparison to NAH. These metabolites are important components of the cell membrane and have been implicated to play a role in cell signalling in diabetes. These findings demonstrate the potential role of phosphatidylcholine and sphingomyelins in IAH.

## Introduction

Impaired awareness of hypoglycaemia (IAH) is characterised by a decrease or absence of the classical sympathoadrenal response and its accompanying symptoms due to a low blood glucose level—hypoglycaemia—such as sweating, shaking, hunger^1^. Warning symptoms that would otherwise prompt an individual to take action, such as consuming extra carbohydrates, are no longer present for a timely response to an oncoming hypoglycaemic event. Consequently, individuals with IAH have a sixfold increased risk of a severe hypoglycaemic event whereby an individual has an altered mental or physical status that requires external aid to restore blood glucose levels^2,3^.

IAH is prevalent in 20–40% of individuals with type 1 diabetes^2,4^. The main risk factor for IAH is antecedent hypoglycaemias^5,6^. Clamp-induced hypoglycaemias in individuals before and after intensive insulin therapy demonstrated significant differences in epinephrine response, as well as in the glucose threshold levels that elicits the epinephrine response^7^. Although it is clear that the counter-regulatory response to a hypoglycaemia is primarily affected in individuals with IAH, the specific pathways that are involved are not completely understood^5,8^. Currently, it is believed that the development of IAH is caused by adaptive changes in the brain. Such adaptations include an increase in glucose transport, alternate fuel use, in combination with potential changes in peripheral and central glucose sensing^5,8,9^.

With the rise of high-throughput technology, metabolomics has become an increasingly popular and affordable method, to not only diagnose disease but also to gain insight in disease mechanisms^10^. Targeted metabolomics allows for the absolute quantification of well-defined metabolites, that have been validated and can be reproduced^11^. In combination with other -omics data, this systems biology approach can help to create a holistic picture of mechanisms involved in IAH. In this study, we therefore used this novel approach as a hypothesis-generating tool. We used targeted metabolomics in combination with genome wide association analyses of metabolites to identify metabolites that may be expressed differentially in individuals with type 1 diabetes, with or without IAH. Differentially expressed metabolites were further investigated in relation to potentially affected biological pathways, to generate new hypotheses on IAH pathophysiology.

## Methods

**Figure 1:**
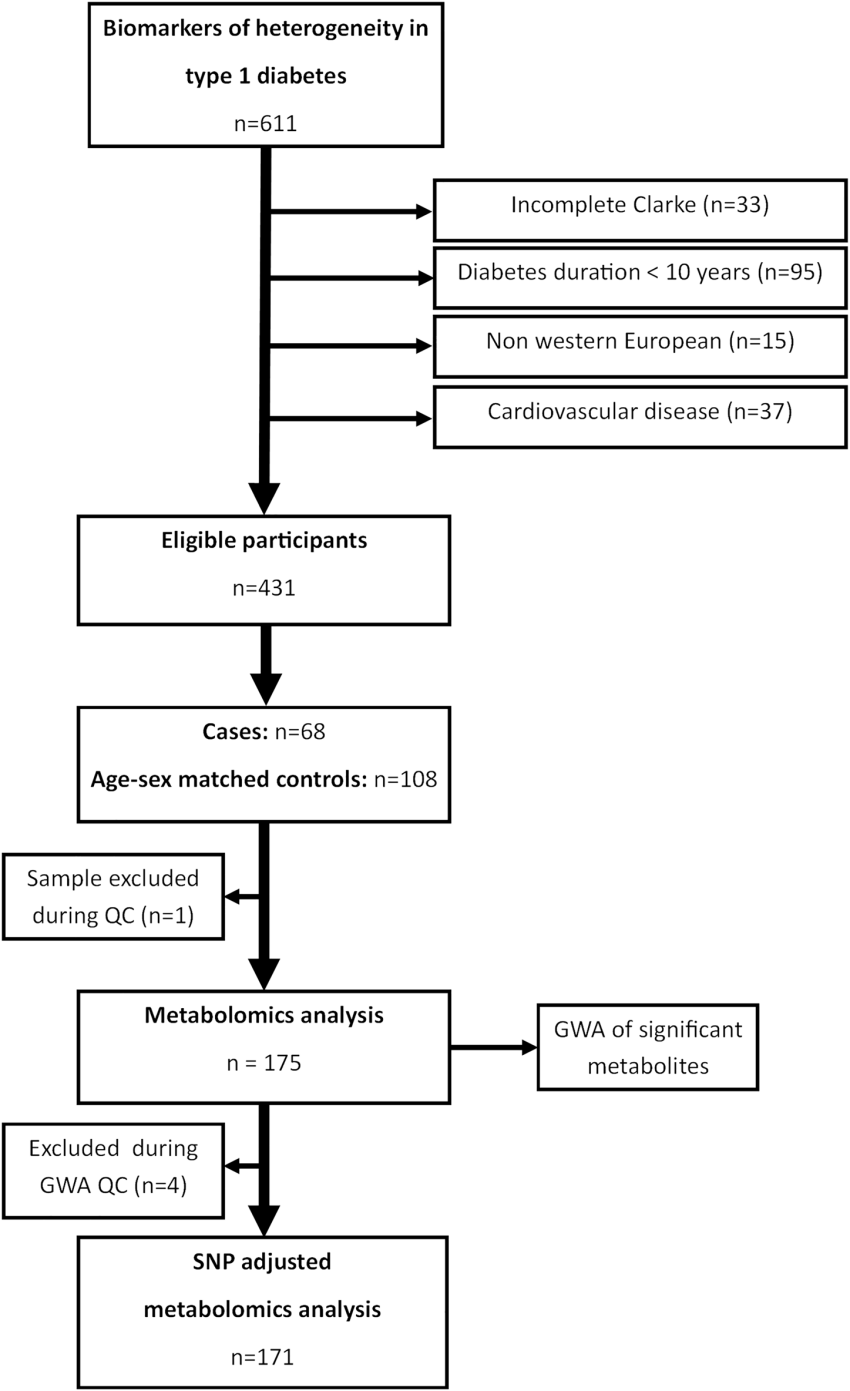
Flow chart of participant inclusion and exclusion, and analyses.

In this nested case–control study, metabolite profiles of individuals with type 1 diabetes and impaired awareness of hypoglycaemia were compared to sex- and age-matched controls with type 1 diabetes but without impaired awareness of hypoglycaemia (Fig. 1). Subsequently, genome wide association studies (GWAS) of the most significant metabolites were conducted. Significant SNPs from the GWAS of metabolites were then re-introduced into the analysis of metabolite profiles between individuals with and without IAH as covariates.

### Participants

Cases and controls were selected from the Dutch type 1 diabetes Biomarkers study (NCT04977635) which ran from 2015 until 2021 in the University Medical Center Groningen, Diabeter, Haaglanden Medical Center, and Ikazia Hospital. This cohort has been described previously in more detail^12^. In short, individuals with type 1 diabetes above the age of 16 years with a diabetes duration greater than 5 years underwent an extensive evaluation between 2015 and 2021 and were prospectively followed. At baseline, 1-year and 2-year follow-up, blood and urine samples, anthropometric measurements, and standardized questionnaires were completed. For this study, data collected during the baseline visit were used.

This study was approved by the medical ethical review committee of the University Medical Center Groningen, Groningen, the Netherlands. Informed consent was obtained from all the participants, in accordance with the declaration of Helsinki.

Individuals were included if they had completed the Dutch clamp-validated Clarke questionnaire^13^ and were Western European, either self-reported or determined by parental place of birth. Exclusion criteria were a diabetes duration < 10 years, as IAH is more prevalent in individuals with longer diabetes duration. Moreover, those with cardiovascular disease as defined as the presence of myocardial infarction, cerebral vascular accident or transient ischaemic attack, and peripheral arterial disease were excluded, as this is known to influence metabolic profiles^14^.

The presence or absence of impaired awareness of hypoglycaemia was determined by a score of ≥ 3 on the Clarke questionnaire (maximum score = 5). Cases were matched to sex- and age-matched controls using nearest neighbour matching with a ratio of 1:1.6, using the MatchIt package in R.

### Metabolite quantification

Blood samples were collected in 10 ml EDTA BD Vacutainer after an overnight fast, when possible. Whether the samples were fasted were determined by self-report during the study visit. Samples were centrifuged at 1300 g for 10 min and the supernatants were aliquoted into 2 ml tubes and stored at -80 °C until use. Serum samples selected for analysis were transported on dry ice to the Helmholtz Institute and further stored at −80 °C until thawed for analysis. Using these reference plasma samples, the long-time stability of plasma metabolites during storage at −80 °C and the performance of the p180 assay have been previously evaluated^15^.

Targeted metabolomics measurements were performed using liquid chromatography- and flow injection-electrospray ionization-tandem mass spectrometry (LC- and FIA-ESI–MS/MS) and the Absolute*IDQ* p180 Kit (BIOCRATES Life Sciences AG, Innsbruck, Austria). The assay allows simultaneous quantification of 188 metabolites out of plasma or serum. The complete assay procedures have been previously published^16^. In brief, 10 µL serum samples were placed into the cavities of the 96-well filter plate of the p180 assay and dried in a nitrogen stream for 30 min. Amino acids and biogenic amines were derivatized with an excess of 5% phenylisothiocyanate for 20 min with a following drying step. Samples were extracted for 30 min at RT with 300 µL methanol containing 5 mM ammonium acetate. The LC run was performed using an Agilent XDB-C18 column (3 × 100 mm, 3.5 µm). Sample handling was performed by a Hamilton Microlab STAR robot (Hamilton Bonaduz AG, Bonaduz, Switzerland) and a Ultravap nitrogen evaporator (Porvair Sciences, Leatherhead, U.K.), besides standard laboratory equipment. Mass spectrometric analyses were done on an API 4000 triple quadrupole system (SCIEX Deutschland GmbH, Darmstadt, Germany) equipped with a 1260 Series HPLC (Agilent Technologies Deutschland GmbH, Böblingen, Germany) and a HTC-xc PAL auto sampler (CTC Analytics, Zwingen, Switzerland) controlled by the software Analyst 1.6.2. For the LC-part, compounds were identified and quantified based on scheduled multiple reaction monitoring measurements (sMRM), for the FIA-part on MRM. Data evaluation for quantification of metabolite concentrations and quality assessment were performed with the software MultiQuant 3.0.1 (SCIEX) and the Met*IDQ* software package, which is an integral part of the Absolute*IDQ* Kit. Metabolite concentrations were calculated using internal standards and reported in µmol/L (µM).

In addition to the study samples, five aliquots of a pooled human reference plasma were analysed on each kit plate.

### Metabolite quality control and normalization

Metabolites in the assay with more than 40% missing data were excluded from analysis. If data were missing in less than 40% metabolite concentrations were imputed by using the minimum value, specified by the kit limit of detection (LOD), divided by the square root of 2, with a random permutation algorithm. Furthermore, metabolites with a coefficient of variance greater than 25% were considered unreliable and removed from the analysis.

Batch normalization was conducted by calculating the plate mean per each metabolite, and then using these means, calculating an overall mean of the three plates. Plate factors were calculated using the overall mean divided by the plate mean, and these plate factors were used as a normalization factor per plate and metabolite. These normalised values were used for further analysis.

### Genotyping, quality control and imputation

Samples collected at baseline measurement were isolated and genotyped using the Infinium GSA array-24 chips v1 and v3 Illumina Inc. (San Diego, USA). Samples that did not meet the following quality control criteria were excluded: sample call rate < 0.95, SNP call rate < 0.96, MAF < 0.01, sex chromosome heterozygosity (between 0.2 and 0.8), identity by descent > 0.185, and samples with a heterozygosity greater or smaller than mean ± 3 SD.

Imputation was conducted using the Minimac4 tool from the Michigan Imputation Server and imputed to the Haplotype Reference Consortium version 1.1^17^. The data was phased using Eagle v2.4 using the HRC1.1 2016 hg (GRCh37/hg19) reference panel. After imputation, variants with MAF < 0.05, imputation quality score of R^2^ < 0.5, and HWE p values < 10^–12^ were filtered.

### Statistical analysis

Descriptive statistics were conducted using R statistical software. Population characteristics are presented as means with standard deviations, medians with 1st and 3rd quartile, and counts with percentages. Statistical differences between those with and without IAH were analysed with either Student t-test, Man-Whitney U test, and Chi-square test where appropriate.

In total, 153 metabolite compounds and 25 sums and ratios of metabolites passed quality control and were included in the analysis. All analyses were conducted using MetaboAnalyst 5.0^18^. The data was first normalised by subtracting the median, transformed using log base 10, and scaled using Pareto scaling^19^. Differential expression was calculated by log2 fold change. Differences in metabolite abundance between NAH and IAH were analysed using the MetaboAnalyst 5.0 platform^18^. Linear models were fitted for each metabolite adjusted for sex, diabetes duration, and fasting glucose. Missing covariate data were replaced by the total population mean. Diabetes duration was used as a covariate instead of age, as these two variables are strongly correlated, and diabetes duration is a more clinically relevant risk factor for IAH. Moreover, a sensitivity analysis was conducted excluding data from individuals with c-peptide > 300 pmol/L, and with antihypertensive or lipid lowering medication use.

Genome wide association models were run using PLINK 2.0. Linear models adjusted for sex and age were fitted using the whole sample population for each significant metabolite. Data were visualised in Manhattan and QQ plots per tested metabolite. We also assessed whether earlier reported SNPs were significantly associated with the specific metabolites in these participants.

In the second metabolite analysis, the genome wide significant SNPs were introduced as covariates in the linear models together with age, diabetes duration and fasting glucose. Metabolites still showing significance after adjusting for SNPs are likely associated with IAH despite the SNP variant associated with higher/lower concentrations of the metabolites.

Metabolites reaching unadjusted p-values of < 0.05 were looked up in the small molecule pathway database (SMPDB) and searched manually in literature.

## Results

In total, 68 cases and 108 sex and age matched controls were selected for the metabolomics analysis (Fig. 1). Table 1 shows the clinical characteristics of the cases and controls.

**Table 1:** Table of characteristics means (SD), median [IQR], and count n (%).

Characteristics	NAH (n = 108)	IAH (n = 67)	*p*-Value
Age, in years	31 [22, 54]	50 [38, 59]	< .001
Age at diagnosis in years	10 [5, 17]	19 [10, 28]	< .001
Sex, male	47 (44)	24 (36)	0.40
Diabetes duration, in years	21 [14, 32]	25 [20, 38]	0.005
BMI, kg/m^2^	26.4 (4.0)	27 (3.7)	0.21
CSII = Yes (%)	67 (62)	41 (61)	1.00
Daily insulin dosage in IE per day	54 [42, 68]	44 [36, 57]	0.03
HbA1c (mmol/l)	62 (13)	60 (11)	0.31
Fasting glucose, mmol/L	9.6 (4.5)	10.1 (3.7)	0.47
C peptide, pmol/L	0 [0, 0]	0 [0, 0]	0.92
Microvascular complications, yes (%)	30 (68)	37 (77)	0.47
Creatinine, (umol/l)	72 [62, 81]	69 [61, 79]	0.28
eGFR, ml/min/1.73m^2^	99 [81, 111]	91 [82, 101]	0.20
Albumin creatinine ratio, mg/mmol	0.69 [0.41, 1.34]	0.60 [0.27, 1.30]	0.18
Systolic blood pressure, mmHg	128 (12)	130 (14)	0.42
Diastolic blood pressure, mmHg	73 (8)	73 (8)	0.95
Antihypertensive medication	20 (19)	22 (33)	0.05
Lipid lowering medication	20 (19)	23 (35)	0.03

*ACR* albumin creatinine ratio, *BMI* Body mass index, *CSII* Continuous subcutaneous insulin infusion, *eGFR* estimated glomerular filtration rate, *DBP* diastolic blood pressure, *SBP* systolic blood pressure. Data were missing for BMI (n = 7), daily insulin dosage (n = 4), HbA1c (n = 1), fasting glucose (n = 1), C peptide (n = 1), microvascular complications (n = 83), eGFR (n = 2), ACR (n = 5), SBP (n = 6), DBP (n = 5), antihypertensive medication (n = 2), lipid lowering medication (n = 1).

Despite attempting to match for age using nearest neighbours, individuals with IAH were significantly older and had a longer diabetes duration than those without. No differences were present in sex, BMI or continuous subcutaneous insulin infusion use. Daily insulin dosage was significantly lower in individuals with IAH, however, HbA1c was comparable between these two groups. Moreover, microvascular complications and kidney function were comparable between individuals with NAH and IAH. Additionally, antihypertensive medication and lipid lowering medication use differed between the groups.

### Metabolomics I

**Figure 2:**
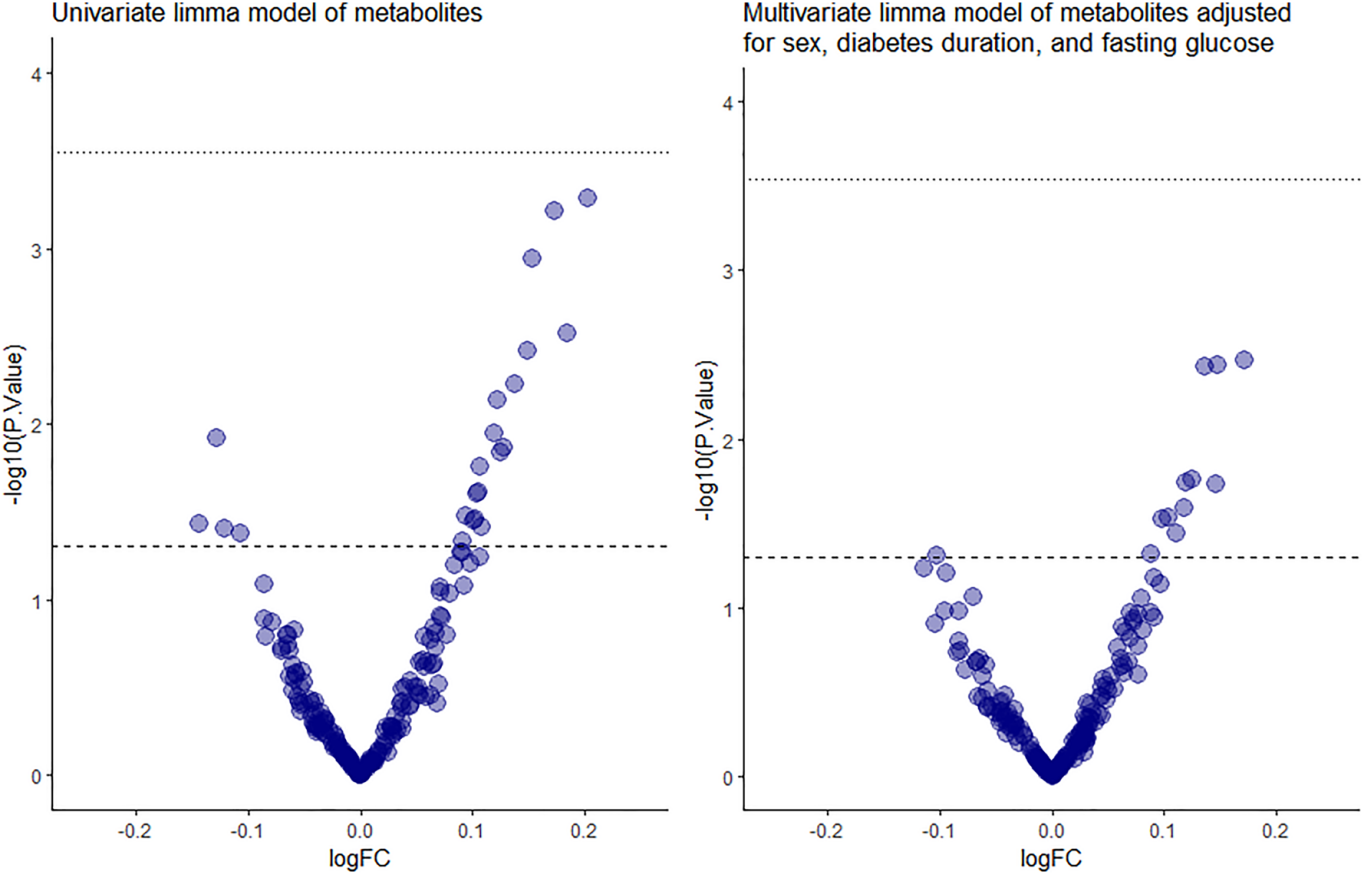
Volcano plot of metabolite magnitude of change. (**A**) Univariate linear model and (**B**) sex, diabetes duration and fasting glucose adjusted model.

From the 188 metabolites and sums/ratios calculated, 175 metabolites passed quality control and were included in the analysis. Univariate linear models led to 22 significant metabolites shown in Fig. 2. Detailed results are given in Supplementary Table 1. After adjusting for sex, log_10_(diabetes duration), and fasting glucose, 12 metabolites remained significant (Table 2). After Benjamini–Hochberg false discovery rate (FDR) adjustment these metabolites were no longer significant. Sensitivity analysis excluding individuals with C-peptide > 300 pmol/L showed no major differences in the metabolomics analysis (Supplementary Table 1).

**Table 2:** Top hits of the metabolomics I, linear models adjusted for sex, log(diabetes duration), and fasting glucose.

Metabolites	logFC	*P* value	Adjusted *P* values	HMDB ID	IAH	NAH
PC.aa.C36.6	0.17	0.003	0.22	HMDB0008690	0.62 ± 0.26	0.52 ± 0.26
PC.ae.C38.0	0.15	0.004	0.22	HMDB0013419	1.60 ± 0.53	1.38 ± 0.54
SM(OH)C24.1	0.14	0.004	0.22	HMDB0013469	1.53 ± 0.38	1.34 ± 0.33
PC.aa.C36.0	0.12	0.02	0.56	HMDB0007886	2.06 ± 0.66	1.78 ± 0.57
SM.C26.1	0.12	0.02	0.56	HMDB0007984	0.46 ± 0.13	0.40 ± 0.11
PC.aa.C36.5	0.15	0.02	0.56	HMDB0013461	21.6 ± 12.0	17.8 ± 10.0
SM.C26.0	0.12	0.03	0.60	HMDB0013433	0.22 ± 0.05	0.20 ± 0.05
PC.ae.C40.1	0.10	0.03	0.60	HMDB0011698	1.25 ± 0.31	1.14 ± 0.32
SM(OH)C22.1	0.10	0.03	0.60	HMDB0013466	14.0 ± 3.15	13.0 ± 2.93
PC.aa.C42.6	0.11	0.04	0.64	HMDB0008734	0.37 ± 0.11	0.33 ± 0.14
PC.ae.C40.2	0.09	0.05	0.74	HMDB0013437	1.37 ± 0.33	1.23 ± 0.31
C16 (Hexadecanoylcarnitine)	−0.10	0.05	0.74	HMDB0000222	0.10 ± 0.03	0.11 ± 0.03

### GWAS metabolites

GWAS of the top 12 metabolites (*p* < 0.05) resulted in 2 models with SNPs reaching genome wide significance. Detailed information is provided in Supplementary Table 2. On chromosome 1, rs2071499 (G > A) was found to be genome wide significant for the metabolite sphingomyelin (d18:0/24:1(15Z)(OH)) (Supplementary Fig. 1). This SNP codes for a missense variant (NP_291031.2:p.Ser7Phe) of *GUCA2A,* which is an endogenous activator of intestinal guanylate cyclase. In the GWAs model for PCaaC36.6, the intergenic SNP rs2876585 (C > T), on chromosome 6 reached genome wide significance (Supplementary Fig. 2).

### Metabolite analysis II

The 2 genome-wide significant SNPs, rs2071499 and rs2876585, were included as covariates in the sex, diabetes duration and glucose adjusted linear metabolite models. In total, 11 metabolites were significant before FDR adjustment (Table 3). After adjusting for multiple testing, no metabolites remained significant.

**Table 3:** Top hits from the metabolomics analysis II, linear models adjusted for sex, log(diabetes duration), fasting glucose, and SNPs rs2071499 and rs2876585.

Metabolites	logFC	*P* value	Adjusted *P* values	HMDB ID	IAH	NAH
PC.ae.C38.0	0.15	0.003	0.22	HMDB0013419	1.60 ± 0.53	1.38 ± 0.54
PC.aa.C36.6	0.16	0.004	0.22	HMDB0008690	0.62 ± 0.26	0.52 ± 0.26
SM(OH) C24.1	0.14	0.004	0.22	HMDB0013469	1.53 ± 0.38	1.34 ± 0.33
PC.aa.C36.0	0.13	0.02	0.61	HMDB0007886	2.06 ± 0.66	1.78 ± 0.57
SM.C26.1	0.12	0.02	0.61	HMDB0013461	0.46 ± 0.13	0.40 ± 0.11
PC.aa.C36.5	0.14	0.02	0.61	HMDB0007984	21.6 ± 12.0	17.8 ± 10.0
SM.C26.0	0.12	0.03	0.61	HMDB0011698	0.22 ± 0.05	0.20 ± 0.05
PC.ae.C40.1	0.10	0.03	0.61	HMDB0013433	1.25 ± 0.31	1.14 ± 0.32
PC.ae.C38.6	0.11	0.04	0.61	HMDB0013409	5.85 ± 1.74	5.24 ± 1.42
PC.aa.C42.6	0.11	0.05	0.61	HMDB0008734	0.37 ± 0.11	0.33 ± 0.14
PC.aa.C42.2	0.11	0.05	0.61	HMDB0008795	0.28 ± 0.10	0.25 ± 0.09
SM(OH)C22.1	0.09	0.05	0.61	HMDB0013466	14.0 ± 3.15	13.0 ± 2.93

### Pathway analysis

The top 11 metabolites (unadjusted p < 0.05) were looked-up in the small molecule pathway database (SMPDB). Two metabolites were described in the SMPDB and were involved in the phosphatidylethanolamine and phosphatidylcholine biosynthesis pathways. Manual search in the literature identified another pathway, the sphingomyelin synthase pathway (Reactome: R-HSA-429786).

## Discussion

In this study, we found that individuals with IAH had higher expression of certain sphingomyelins and glycerophospholipids compared to those with normal awareness of hypoglycaemia. Despite these differences no longer being significant after correction for multiple testing, these metabolite groups remained differentially expressed after adjusting for confounders, including SNPs associated with metabolite expression. These metabolites included PC ae C38:0, PC aa C36:6, SM(OH) C24:1, PC.aa.C36.0, SM.C26.1, PC.aa.C36.5, SM.C26.0, PC.ae.C40.1, PC.ae.C38.6, PC.aa.C42.6, PC.aa.C42.2, and SM(OH)C22.1. SM(OH)C22:1 and PC 36:6 had genome-wide significant SNPs associated with their expression, which have previously been described in other metabolite GWAS studies.

Sphingomyelins are primarily found in mammalian cell membranes, particularly in the myelin sheaths which surround nerve cell axons which is essential for nerve functioning. Sphingomyelin consists of a ceramide core and a phosphatidylcholine group, and its synthesis involves the transfer of phosphorylcholine from phosphatidylcholine to ceramide resulting in the release of diacylglycerol^20,21^. The synthesis of sphingomyelin is regulated by the enzyme sphingomyelin synthase, and the reverse reaction is regulated by sphingomyelinase^21^. A well characterised disease involving an aberration in this pathway is Niemann-Pick type A Disease, in which there is a deficiency of acid sphingomyelinase leading to an accumulation of sphingomyelin and its precursors^22^. This accumulation of precursors and sphingomyelin results in neurovisceral manifestations with neural symptoms such as vertical supranuclear gaze palsy, cerebellar ataxia, and progressive dementia illustrating the importance of this pathway in nerve functioning^22^.

In addition to the structural importance of sphingomyelin in the cell membrane, it has been found to contribute to cellular events through signalling of cell growth, differentiation, inflammation, and apoptosis^21,23–25^. Previous research has shown higher circulating levels of ceramides after hypoglycaemia, as a result of acid sphingomyelinase hydrolysing sphingomyelin into ceramide and phosphorylcholine. The high levels of ceramides have been shown to induce neuronal death, and the inhibition of sphingomyelinase has been shown to reduce neuronal death^26^. Moreover, sphingolipids have been extensively studied in disease pathology, including insulin resistance and type 2 diabetes^21^. Circulating plasma ceramides are higher amongst individuals with type 2 diabetes and studies have demonstrated an improvement in insulin signalling when ceramide degradation is stimulated^27,28^. These effects are a result of the inhibition of phosphatidylinositol-3 kinase (PI3K) and the blocking of AKT/PKB, which are important in insulin signalling and GLUT4 translocation^28^. In our study we observed higher levels of sphingomyelins and phosphatidylcholine in individuals with IAH. This could suggest that either the synthesis of sphingomyelin or the degradation of ceramides may be upregulated, which would result in a net lowering of ceramides. Recurrent hypoglycaemia’s may blunt the previously mentioned increase in circulating ceramides, leading to less neuron death. Looking into the expression of key enzymes such as sphingomyelin synthase or sphingomyelinase before and after recurrent hypoglycaemias would give a better understanding of the potential adaptive changes in these pathways leading to IAH.

Phosphatidylcholine is the most prevalent phospholipid in cell membranes and subcellular organelles, such as mitochondria and lipoproteins^29^. Similar to the sphingolipids, phosphatidylcholine plays an integral role in regulating metabolism, and disbalances have been implicated in the pathophysiology of type 2 diabetes and insulin signalling^30,31^.

In our study we found higher levels of phosphatidylcholine (PC aa C36:6, PC aa C36:0, PC aa C36:5, PC aa C42:6, PC ae C38:0, PC ae C40:1, PC ae C40:2) in individuals with IAH in comparison to those with NAH. A previous study demonstrated that higher PC aa C38:3 levels were important in the transition to type 2 diabetes^30^. Higher levels of ceramides can inhibit the insulin pathway through the activation of the aryl hydrocarbon receptor which inhibits the *IRS-2* gene^30^. Increase in insulin resistance could be beneficial in individuals with IAH, as the recurrent hypoglycaemias are a result of the iatrogenic hyperinsulinemia^32^, and an increased insulin resistance may therefore lead to less hypoglycaemia under the same insulin dose^33^. However, in our study population we found that those with IAH had comparable HbA1c despite less daily insulin use, suggesting these individuals are more insulin sensitive. Whether these are individuals at greater risk for IAH, or whether this is a compensatory mechanism, is still to be determined. Further studies are necessary to elucidate the impact of insulin resistance or sensitivity in individuals with IAH.

Another potential explanation for the higher levels in individuals with phosphatidylcholine is a difference in diet. High fat diets are known to induce overproduction of phosphatidylcholine, and dietary patterns may potentially confound findings^34,35^. Nevertheless, our findings suggest that phosphatidylcholines may be of further interest to investigate in the pathophysiology or repercussions of IAH. However, future research should consider measures of insulin resistance and diet to eliminate potential confounding.

Genome wide significant SNPs, rs2071499 and rs2876585, were found for the level of the metabolites SM(OH) C22:1 and PC aa C36:6 in our study. These SNPs have not yet been described in large metabolite GWASs to date^36–38^. Post-hoc power calculation of the genetic signals on SM (OH) C22:1 and PC aa C36:6 in our study showed a power of 99%. This may potentially indicate a type 1 diabetes specific signal. In previous GWAs of metabolites using the Biocrates panel, the SNP rs445925 on chromosome 19 and rs102275 on chromosome 11 were found to be significantly associated with SM (OH) C22:1 and PC 36:6, respectively. A potential reason for previously described SNPs not being found in our study is the smaller sample size. In order to have 80% power to detect these signals, we would require a sample size of between 3800–4500 participants. Rs2071499 is found in *GUCA2A*, which codes for guanylin; an activator of intestinal guanylate cyclase^39^. Guanylin peptides are important in electrolyte reabsorption, and mutations are associated with adenocarcinomas and polyps^40^. Studies have described the potential importance of *GUCA2A* in intestinal injury and inflammation^39,41^. However, no studies have yet described any association with metabolite concentrations.

### Strengths and limitations

In this study we describe the metabolic profile of IAH in individuals with type 1 diabetes. Adjustment with both environmental and genetic confounders, lead to consistent results reaching near significant trends. Moreover, we found novel GWA signals of metabolites in individuals with type 1 diabetes, with SNPs reaching genome wide significance with higher power, despite a relatively small sample size for GWAS.

Limitations of this study include the cross-sectional design and the inability to study the environmental influences on the metabolites and IAH. IAH is dynamic, with some individuals regaining awareness of hypoglycaemias after treatment and avoidance of recurrent hypoglycaemic episodes. Although the Clarke questionnaire takes the episodes of moderate and severe hypoglycaemias into consideration, the impact that these episodes have on metabolites in the short- and long term cannot be disentangled in this study. Specifically, investigating metabolites in euglycemic, hypoglycaemic and in controlled states of IAH will enable better pinpointing of pathways involved^42^. Moreover, investigating different glycaemic states in healthy controls may determine any normal responses to hypoglycaemia habituation and IAH specific adaptation. Nonetheless, this study has been able to identify potential pathways that can be further investigated experimentally.

In conclusion, glycerophospholipids and sphingomyelins appeared to be associated with IAH, and two SNPs were found to be significantly associated with the metabolites SM (OH) C22:1 and PC aa C36:6. Future studies in the relationship between ceramides, phosphatidylcholines, and sphingomyelins may be interesting to investigate in the context of IAH.

### Supplementary Information

Supplementary Information.

## Data Availability

The data that support the findings of this study are available from the Dutch type 1 diabetes Study group, but restrictions apply to the availability of these data, which were used under license for the current study, and so are not publicly available. Data are however available from the corresponding authors upon reasonable request and with permission of bwo@umcg.nl.

## Abbreviations

FDR: False discovery rate
GWAS: Genome wide association study
IAH: Impaired awareness of hypoglycaemia
LOD: Limit of detection
NAH: Normal awareness of hypoglycaemia
