# Supplementary material for: Metabolomic associations of impaired awareness of hypoglycaemia in type 1 diabetes
Source: Sci Rep. 2024 Feb 23;14:4485. doi: 10.1038/s41598-024-55032-6 (PMC10891160; doi:10.1038/s41598-024-55032-6)

# Supplementary materials

**Supplementary table 1: Sensitivity analysis excluding individuals with C-peptide >300 pmol/L**

|  | logFC | P value | Adj P value | HMDB ID |
| --- | --- | --- | --- | --- |
| SM OH C24:1 | 0.15 | 0.001 | 0.20 | HMDB0013469 |
| PC ae C38:0 | 0.15 | 0.004 | 0.31 | HMDB0013419 |
| PC aa C36:6 | 0.17 | 0.01 | 0.31 | HMDB0008690 |
| SM C26:1 | 0.13 | 0.01 | 0.31 | HMDB0013461 |
| PC aa C36:0 | 0.14 | 0.01 | 0.31 | HMDB0007886 |
| SM C26:0 | 0.13 | 0.02 | 0.31 | HMDB0011698 |
| SM OH C22:1 | 0.11 | 0.03 | 0.31 | HMDB0013466 |
| PC ae C40:1 | 0.11 | 0.03 | 0.51 | HMDB0013433 |
| PC ae C36:5 | 0.14 | 0.03 | 0.51 | HMDB0007984 |
| PC aa C42:6 | 0.12 | 0.03 | 0.51 | HMDB0008734 |
| PC ae C40:2 | 0.10 | 0.03 | 0.54 | HMDB0013437 |
| PC ae C40:6 | 0.09 | 0.05 | 0.65 | HMDB0013422 |

**Supplementary table 2: GWAS results**

| Metabolite | SNP | rs | B | SE | P | MAF |  |
| --- | --- | --- | --- | --- | --- | --- | --- |
|  |  |  |  |  |  |  |  |
| PC aa C36:6 | 6:144594852_C/T | rs2876585 | -0.2724 | 0.0465 | 4.6 x 10-9 | 0.40 | unadjusted |
|  |  |  | -02463 | 0.0433 | 1.2 x 10-8 |  | adjusted for sex and age |
|  |  |  |  |  |  |  |  |
| SM OH C22:1 | 1:42630364_G/A | rs2071499 | 0.1370 | 0.0236 | 6.7 x 10-9 | 0.37 | unadjusted |
|  |  |  | 0.1292 | 0.0231 | 2.1 x 10-8 |  | adjusted for sex and age |

Supplementary figure 1: Locuszoom plot of significant SNP in the GWAs of SM(OH)C22:1


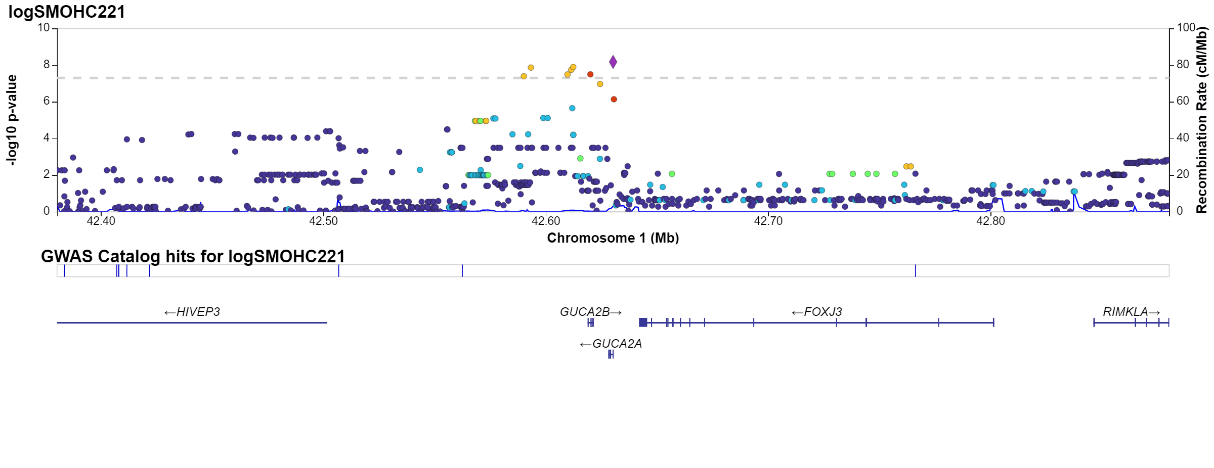


**Supplementary figure 2:** Locuszoom plot of significant SNP in the GWAs of PC C36:6


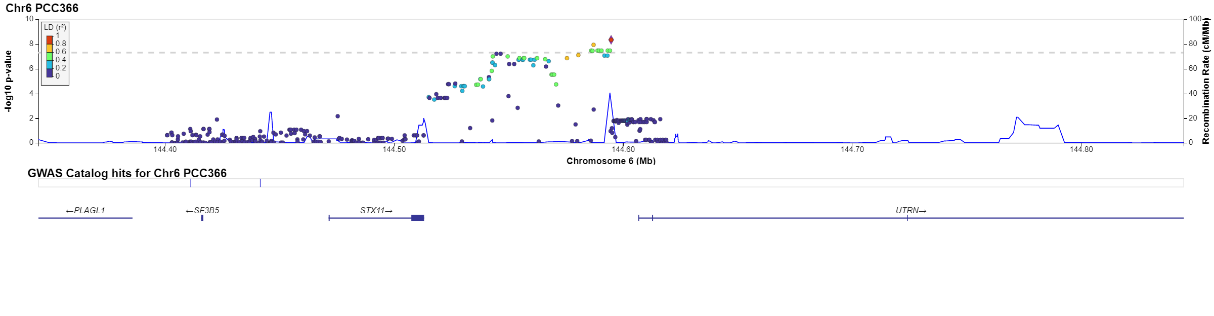

Supplement: Supplementary file 1 — Supplementary Information. [file 41598_2024_55032_MOESM1_ESM.docx]
